# Novel X-ray Communication Based XNAV Augmentation Method Using X-ray Detectors

**DOI:** 10.3390/s150922325

**Published:** 2015-09-03

**Authors:** Shibin Song, Luping Xu, Hua Zhang, Yuanjie Bai

**Affiliations:** 1 School of Aerospace Science and Technology, Xidian University, Xi’an 710126, China; E-Mails: songshibin@stu.xidian.edu.cn (S.S.); zhanghua@mail.xidian.edu.cn (H.Z.); 2 Xi’an Microelectronics Technology Institute, Xi’an 710054, China; E-Mail: byjck666@163.com

**Keywords:** XNAV augmentation, X-ray communication, X-ray ranging, X-ray detector, measurement model

## Abstract

The further development of X-ray pulsar-based NAVigation (XNAV) is hindered by its lack of accuracy, so accuracy improvement has become a critical issue for XNAV. In this paper, an XNAV augmentation method which utilizes both pulsar observation and X-ray ranging observation for navigation filtering is proposed to deal with this issue. As a newly emerged concept, X-ray communication (XCOM) shows great potential in space exploration. X-ray ranging, derived from XCOM, could achieve high accuracy in range measurement, which could provide accurate information for XNAV. For the proposed method, the measurement models of pulsar observation and range measurement observation are established, and a Kalman filtering algorithm based on the observations and orbit dynamics is proposed to estimate the position and velocity of a spacecraft. A performance comparison of the proposed method with the traditional pulsar observation method is conducted by numerical experiments. Besides, the parameters that influence the performance of the proposed method, such as the pulsar observation time, the SNR of the ranging signal, *etc.*, are analyzed and evaluated by numerical experiments.

## 1. Introduction

Being an autonomous navigation method applied for solar system and beyond, XNAV (X-ray pulsar-based NAVigation) has been a hot research area during the past ten years. Multiple aspects of XNAV, including Time of Arrival (TOA) measurement [1,2,3], time transfer [4,5,6], ambiguity resolution [7,8,9], filtering algorithms [10,11] *etc.*, have been studied to verify the feasibility of XNAV. The theoretical positioning accuracy of XNAV is 10 m. Currently, the available accuracy of XNAV is about several hundred meters, which is far from the designed accuracy [7]. The gap is caused by various factors, including the inaccuracy of the noise model, the relativistic effects, the limitations of current X-ray detectors, the ephemeris error, *etc*. The limited available accuracy greatly hinders the application of XNAV, therefore, how to improve the positioning accuracy of XNAV under current conditions has become a critical and urgent issue.

The main solution of improving XNAV accuracy is to obtain extra information from the available navigation systems or multiple space sources. Reference [12] provided an integration of XNAV with a strap-down inertial system (SINS), which provides higher accuracy and requires shorter filtering period, but the SINS error accumulation would affect the performance of the integrated system, especially for long-duration missions. Another main method to augment XNAV is by observing stars, planets, or asteroids. In the solar system, the Sun is an obvious and readily available source for observation, which is used in XNAV augmentation. In Reference [13], a Sun sensor was utilized to observe the line-of-sight vector towards the Sun and the line-of-sight vector was integrated with X-ray pulsar observation to achieve autonomous navigation in Halo orbit. Reference [14] also utilized the line-of-sight vector of the Sun as the observation variable and adopted a residual orthogonal unscented Kalman filter (ROUKF) for navigation filtering. Planets are also common observation targets in the navigation of satellites that orbit around a central body. Accurate navigation is realized by combining the pulsar observation and the central body observation that is achieved by a star sensor [15,16]. This method of observing a central body has the obvious disadvantage of a limited application range. The method can only be used near the central body. For specific missions, asteroids are used to improve XNAV performance. For example, in Reference [17], the images of asteroids, captured by a navigation camera, were combined with pulsar observation to realize navigation in an interplanetary cruise. This method would have a great dependence on the quality of the asteroid images. Though utilizing the observation of space sources to improve XNAV has shown some potential, both the integration method using a Sun sensor and the augmentation method with central body observation or asteroid observation share the same flaw in that the star sensors have a limited accuracy and easily suffer from interference by noise. Thus, the improvements of XNAV based on the abovementioned methods are limited.

In this paper, we propose an augmentation method for XNAV based on X-ray communication (XCOM), which utilizes accurate X-ray ranging as the extra observation to improve the XNAV accuracy. XCOM is a newly emerged concept for deep space communication [18], which utilizes X-rays as the transmission medium. Being high energy light and not being affected by electrical and magnetic field [19], X-rays show fine space propagation performance. The idea of X-ray ranging is derived from XCOM. In our previous research, we have introduced X-ray ranging and presented a detailed performance analysis [20]. Compared with available augmentation methods, X-rays could provide accurate range measurements, which could serve as the observation variable in XNAV. In other words, by both X-ray pulsar observation and X-ray ranging measurement, the position and velocity of a spacecraft could be estimated by a Kalman filtering algorithm. Actually, there is much more potential in combining XNAV with XCOM. Besides accurate range measurement, XCOM-based simultaneous communication and ranging could also provide communication services [21]. As is known to all, the phase revolution model is of great importance in estimating TOA in XNAV and should be updated periodically [6]. With XCOM-based simultaneous communication and ranging, the phase revolution model could be updated in a timely fashion, which would benefit deep space missions far from the Earth. In a word, XCOM could not only provide the accurate observation variable needed for XNAV augmentation, but also a communication service for the transmission of key information. XCOM would be a great supplement for XNAV and provide the possibility of integrated X-ray navigation and communication for future deep space explorations.

## 2. Experimental Section

### 2.1. Coordinate Systems

To calculate the accurate TOA, the time measurement should be conducted in an inertial coordinate frame. For pulsar observations, the solar system barycenter (SSB) frame is the preferable coordinate. The SSB frame is also called the International Celestial Reference Frame (ICRF), which is an inertial reference system that uses the solar system barycenter as the center of the frame [22].

When in the vicinity of Mars, the Mars-centered coordinate system should be utilized. The fundamental Mars-centered inertial coordinate system is the Mars-centered Earth Mean Equator and Equinox of Epoch [23]. This coordinate is formed by moving the Earth Mean Equator and Equinox of Epoch reference system from the Earth center to the Mars center. The Earth Mean Equator and Equinox of Epoch reference system is defined as follows: (1) the reference plane is set to be the Earth mean equator; (2) the reference direction is set to be the vernal equinox of the Earth; (3) the J2000.0 epoch is selected as the reference epoch. Another commonly used Mars-centered inertial reference system is the Mars-centered Mars Mean Equator and Equinox of Epoch, which is derived from the Mars-centered Earth Mean Equator and Equinox of Epoch. The Mars-centered Mars Mean Equator and Equinox of Epoch is defined as follows: (1) the reference plane is the Mars mean equator of J2000.0; (2) the reference direction is the ascending node of the Mars mean equator relative to the Earth Mean Equator; (3) the reference is set to the J2000.0 epoch. Currently, the Mars-centered Mars Mean Equator and Equinox of Epoch is the most popular reference system in Mars research.

### 2.2. Orbit Dynamics

We define the system state vector as $X={\left[r^{\text{T}},v^{\text{T}}\right]}^{\text{T}}={\left[x,y,z,v_{x},v_{y},v_{z}\right]}^{\text{T}}$, in which $r={\left[x,y,z\right]}^{\text{T}}$ defines the position of the spacecraft relative to the Mars center and $v={\left[v_{x},v_{y},v_{z}\right]}^{\text{T}}$ describes the velocity. Let f(⋅) denote the description function of the spacecraft dynamics. Then, the spacecraft state can be expressed as:

(1) $$\dot{X}=f(X(t))+w(t)$$

where w(t) is the process noise. Let $\tilde{X}(t)$ be the optimal estimation of the system state and define $\delta X=X(t)-\tilde{X}(t)$.

Take the Taylor expansion of Equation (1) with respect to $\tilde{X}(t)$, and we have:

(2) $$\dot{X}=f(\tilde{X}(t))+{\frac{\partial f(X)}{\partial X}|}_{X=\tilde{X}}\delta X+w(t)$$

where the high-order terms are ignored. Then, there is:

(3) $$\begin{array}{l} \delta \dot{X}={\frac{\partial f(X)}{\partial X}|}_{X=\tilde{X}}\delta X+w(t)\\ \;=F(t)\delta X+w(t) \end{array}$$

If the process noise is ignored, f(X(t)) can be expressed by the velocity, v, and the acceleration, a:

(4) $$\begin{array}{l} f(X(t))={\left[{\dot{r}}^{T},{\dot{v}}^{T}\right]}^{\text{T}}\\ \;={\left[v^{\text{T}},a^{\text{T}}\right]}^{\text{T}} \end{array}$$

Then, F(t) can be expressed as:

(5) $$\begin{array}{l} F(t)={\frac{\partial }{\partial X}\left[\begin{array}{l} v\\ a \end{array}\right]|}_{X=\tilde{X}}\\ \;={\left[\begin{array}{cc} 0_{3\times 3} & I_{3\times 3}\\ \frac{\partial a}{\partial r} & \frac{\partial a}{\partial v} \end{array}\right]|}_{X=\tilde{X}} \end{array}$$

where I is the unit matrix.

The decentralized form of Equation (3) can be written as:

(6) $$\delta X_{k}=\Phi_{k}\delta X_{k}+w(t)$$

where $\Phi_{k}$ is the state transition matrix that satisfies:

(7) $$\left\{ \begin{array}{l} \dot{\Phi }(t_{k},t_{0})=F(t_{k})\Phi (t_{k},t_{0})\\ \Phi (t_{0},t_{0})=I \end{array}\right.$$

Generally, $\Phi_{k}$ can be calculated by the Runge-Kutta (RK) method [24]. Considering the perturbations, the acceleration of a spacecraft orbiting the Mars, a, can be written as:

(8) $$\begin{array}{l} a=a_{0}+a_{p}\\ \;=-\mu_{M}\frac{r}{r^{3}}+a_{p} \end{array}$$

where $a_{0}$ is the gravitational acceleration of the Mars, $a_{p}$ is the acceleration caused by various perturbation sources, $\mu_{M}$ is the gravitational constant of the Mars, and r=||r||. The perturbation sources of Mars orbit include the non-spherical perturbation, the third-body perturbation, the solar radiation pressure perturbation, and the atmosphere drag perturbation, *etc.* For the high Mars orbits, the main perturbation sources are the non-spherical perturbation and the third-body perturbation. Other perturbations, such as the atmosphere effect, the solar radiation pressure, *etc.*, are ignored for the high orbit case.

The non-spherical perturbation is caused by the irregularity of the mass distribution and the shape of Mars. The gravitational potential function of Mars can be expressed as [24]:

(9) $$U=\frac{\mu_{M}}{r}\sum_{l=0}^{\infty }\sum_{m=0}^{l}{\left(\frac{R}{r}\right)}^{l}P_{lm}(\sin\varphi )\left(C_{lm}\cos(m\lambda )+S_{lm}\sin(m\lambda )\right)$$

where $P_{nm}(\cdot )$ is the n-order m-degree associated Legendre function, *R* is the average radius of Mars, r is the distance of the spacecraft relative to the Mars center, λ is the longitude of the spacecraft, φ is the reduced latitude. $C_{lm}$ and $S_{lm}$ are the gravitational potential parameters that reflect the mass distribution of Mars, whose values can refer to the Goddard Mars Model: GMM-2B [25]. The acceleration can be expressed as:

(10) $$\ddot{r}=\nabla U$$

where ∇ is the gradient computation. Define the zonal harmonic terms, $J_{l}$ as:

(11) $$J_{l}=-C_{l0}$$

Other gravitational potential parameters are named as the sectorial harmonic terms (m<l) and the tesseral harmonic terms (m=l). J2 perturbation is the main part of the non-spherical perturbation. Thus, the acceleration caused by the non-spherical perturbation can be expressed as:

(12) $$a_{non-spherical}=\left[\begin{array}{c} -\frac{\mu_{M}x}{r^{3}}\left[1+J_{2}{\left(\frac{R_{M}}{r}\right)}^{2}\left(1.5-\frac{7.5z^{2}}{r^{2}}\right)\right]\\ -\frac{\mu_{M}y}{r^{3}}\left[1+J_{2}{\left(\frac{R_{M}}{r}\right)}^{2}\left(1.5-\frac{7.5z^{2}}{r^{2}}\right)\right]\\ -\frac{\mu_{M}z}{r^{3}}\left[1+J_{2}{\left(\frac{R_{M}}{r}\right)}^{2}\left(4.5-\frac{7.5z^{2}}{r^{2}}\right)\right] \end{array}\right]$$

The third-body perturbation is caused by the gravitational force from other celestial bodies, mostly from the Sun and the Earth. The third-body acceleration, denoted by $a_{third-body}$, could be expressed as:

(13) $$a_{third-body}=-\mu_{s}\left(\frac{r_{sp}}{r_{sp}^{3}}-\frac{r_{s}}{r_{s}^{3}}\right)-\mu_{e}\left(\frac{r_{ep}}{r_{ep}^{3}}-\frac{r_{e}}{r_{e}^{3}}\right)$$

where $\mu_{s}$ is the gravitational constant of the Sun, $\mu_{e}$ is the gravitational constant of the Earth, $r_{s}$ is the position vector of the Sun in the Mars inertia coordinate, $r_{sp}$ is the position vector of the Sun relative to the spacecraft, $r_{e}$ denotes the position vector of the Earth in the Mars inertia coordinate, and $r_{ep}$ is the position vector of the Earth relative to the spacecraft. Based on the aforementioned analysis, the perturbation acceleration can be calculated as:

(14) $$a_{p}=a_{non-spherical}+a_{third-body}$$

### 2.3. Measurement Models

As for the proposed XNAV augmentation method, both the pulsar signal and the range measurement obtained based on XCOM are observed. In this section, the observation equations are established.

#### 2.3.1. Pulsar Timing Observation

**Figure 1:**
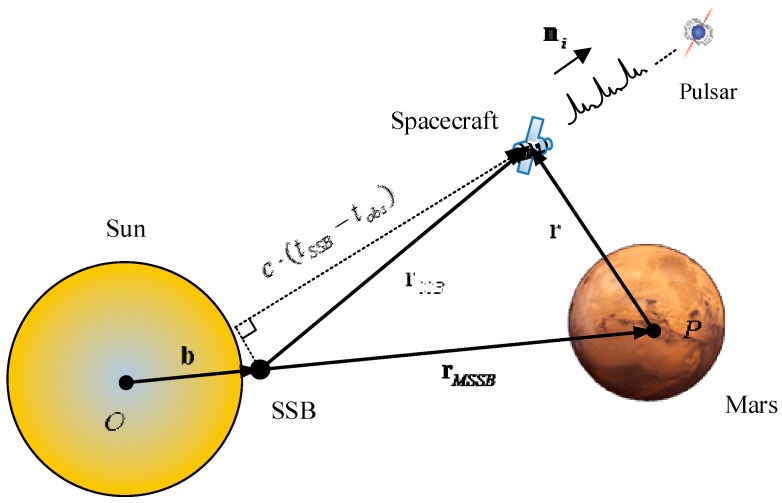
Pulsar timing observation scheme.

Pulsar signals are pulsed period signals with high stability. At the Solar System Barycenter (SSB), a pulsar phase revolution model is established based on the long-duration pulsar observation, which can be utilized to predict the Time of Arrival (TOA) of pulsar pulses at SSB [26]. By observing the TOA of the pulsar signals and comparing the TOA with the time predicted by the phase prediction model [27,28], one can obtain the position of the spacecraft [7]. As the TOA is observed at the spacecraft, it should transferred to Solar System Barycenter (SSB) [7]. During the time transfer, both the geometric effect and other effects, for example, the relativistic effects, should be taken into account. The principle of the time transfer is indicated in Figure 1, where b is the vector pointing from the Sun center to SSB, $r_{SSB}$ is the position of the spacecraft relative to SSB, $r_{MSSB}$ is the position of the Mars center relative to SSB, and $n_{i}$ is the light-of-sight of pulsar signal for the ith observed pulsar. $r_{SSB}$ can be calculated by $r_{SSB}=r_{MSSB}+r$.

Let $t_{obs}$ be the pulse time of arrival at the spacecraft and $t_{SSB}$ be the time of arrival at SSB for the same pulse. Then, the simplified time transfer expression of high orders can be presented as [7]:

(15) $$\begin{array}{l} t_{SSB}=t_{obs}+\frac{n_{i}\cdot r_{SSB}}{c}\\ \text{ +}\frac{1}{2cD_{0}}\left[{\left(n_{i}\cdot r_{SSB}\right)}^{2}-r_{SSB}^{2}+2\left(n_{i}\cdot b\right)\left(n_{i}\cdot r_{SSB}\right)-2\left(b\cdot r_{SSB}\right)\right]\\ \;+\frac{2\mu_{s}}{c^{3}}\ln\left|\frac{n_{i}\cdot r_{SSB}+r_{SSB}}{n_{i}\cdot b+b}+1\right| \end{array}$$

where $D_{0}$ is the range between the barycenter of the Sun and the pulsar, c is the speed of light, $r_{SSB}=\;||r_{SSB}||$, and b=  ||b||.

Based on Equation (15), the pulsar observation can be expressed as:

(16) $$\begin{array}{l} y(t)=c(t_{SSB}-t_{obs})+v(t)\\ \;=n_{i}\cdot r_{SSB}\text{+}\frac{1}{2D_{0}}\left[{\left(n_{i}\cdot r_{SSB}\right)}^{2}-r_{SSB}^{2}+2\left(n_{i}\cdot b\right)\left(n_{i}\cdot r_{SSB}\right)-2\left(b\cdot r_{SSB}\right)\right]\\ \;+\frac{2\mu_{s}}{c^{2}}\ln\left|\frac{n_{i}\cdot r_{SSB}+r_{SSB}}{n_{i}\cdot b+b}+1\right|+v(t)\\ \;=h(X,t)+v(t) \end{array}$$

where h(X,t) is a nonlinear function of X, i denotes the ith pulsar that is observed and $v_{i}$ is the observation noise, which is generally modeled as a white Gaussian noise.

As defined previously, r satisfies $r_{SSB}=r+r_{MSSB}$. Define $\tilde{X}=[{\tilde{r}}^{\text{T}},{\tilde{v}}^{\text{T}}{]}^{\text{T}}$ be the estimation of the system state. Then, the measurement residual can be defined as:

(17) $$\begin{array}{l} z_{p}=y(t)-h(\tilde{X},t)\\ \;=h(X,t)-h(\tilde{X},t)\\ \;={\frac{\partial h(X,t)}{\partial X}|}_{X=\tilde{X}}\delta X+v(t) \end{array}$$

In Equation (17), the first order approximation of h(X,t) is utilized, *i.e.*:

(18) $$h(X,t)=h(\tilde{X},t){+\frac{\partial h(X,t)}{\partial X}|}_{X=\tilde{X}}\cdot (X-\tilde{X})+\varepsilon (t)$$

where ε(t) are the high-order terms that are ignored.

As the estimation of the state is $\tilde{X}=[{\tilde{r}}^{\text{T}},{\tilde{v}}^{\text{T}}{]}^{\text{T}}$, we have:

(19) $$\begin{array}{l} h(\tilde{X},t)=c({\tilde{t}}_{\text{SSB}}-{\tilde{t}}_{\text{SC}})\\ \;=n_{i}\cdot {\tilde{r}}_{\text{SSB}}\\ \;+\frac{1}{2D_{0}}\left[{\left(n_{i}\cdot {\tilde{r}}_{\text{SSB}}\right)}^{2}-{\tilde{r}}_{\text{SSB}}^{\text{2}}+2\left(n_{i}\cdot b\right)\left(n_{i}\cdot {\tilde{r}}_{\text{SSB}}\right)-2\left(b\cdot {\tilde{r}}_{\text{SSB}}\right)\right]\\ \;+\frac{2\mu_{s}}{c^{2}}\ln\left|\frac{n_{i}\cdot {\tilde{r}}_{SSB}+{\tilde{r}}_{SSB}}{n_{i}\cdot b+b}+1\right| \end{array}$$

where ${\tilde{r}}_{SSB}=r_{MSSB}+\tilde{r}$. Then, the measurement residual can be written as:

(20) $$\begin{array}{l} z_{p}=y(t)-h(\tilde{X},t)\\ \;=n_{i}\cdot \delta r\\ \text{ +}\frac{1}{D_{0}}\left[\left(n_{i}\cdot \delta r\right)\left(n_{i}\cdot {\tilde{r}}_{SSB}\right)-{\tilde{r}}_{SSB}\cdot \delta r+\left(n_{i}\cdot b\right)\left(n_{i}\cdot \delta r\right)-b\cdot \delta r\right]\\ \text{ +}\frac{2\mu_{s}}{c^{2}}\left(\frac{n_{i}\cdot \delta r+\frac{{\tilde{r}}_{SSB}}{{\tilde{r}}_{SSB}}\delta r}{n_{i}\cdot {\tilde{r}}_{SSB}+{\tilde{r}}_{SSB}+n_{i}\cdot b+b}\right)+v\\ \;=H_{p}\delta X+v \end{array}$$

where $H_{p}$ is the pulsar observation matrix defined as:

(21) $$H_{p}={\left[\begin{array}{c} \begin{array}{l} n_{i}\text{+}\frac{1}{D_{0}}\left[\left(n_{i}\cdot {\tilde{r}}_{SSB}\right)n_{i}-{\tilde{r}}_{SSB}+\left(n_{i}\cdot b\right)n_{i}-b\right]\\ \text{+}\frac{2\mu_{s}}{c^{2}}\left(\frac{n_{i}+\frac{{\tilde{r}}_{SSB}}{{\tilde{r}}_{SSB}}}{n_{i}\cdot {\tilde{r}}_{SSB}+{\tilde{r}}_{SSB}+n_{i}\cdot b+b}\right) \end{array}\\ 0\\ 0\\ 0 \end{array}\right]}^{\text{T}}$$

and v is the pulsar observation noise. The decentralized form of Equation (20) can be expressed as:

(22) $$z_{p,k}=H_{p,k}\delta X_{k}+v_{k}$$

As to the pulsar timing observation, the observation noise is related to the pulsar parameters:

(23) $$\begin{array}{l} SNR=\frac{F_{x}Ap_{f}t_{obs}}{\sqrt{\left[B_{x}+F_{x}(1-p_{f})\right](At_{obs}d)+F_{x}Ap_{f}t_{obs}}}\\ \;=\frac{F_{x}p_{f}\sqrt{At_{obs}}}{\sqrt{\left[B_{x}+F_{x}(1-p_{f})\right]d+F_{x}p_{f}}} \end{array}$$

(24) $$\sigma_{v_{i}}=\frac{\frac{1}{2}W}{SNR}$$

where $\sigma_{v_{i}}$ is the standard variance of the timing measurement noise, $F_{x}$ is the flux intensity, $B_{X}$ is the background radiation flux, $p_{f}$ is the pulsed fraction that defines the percentage of the source flux that is pulsed, $t_{obs}$ is the observation interval, A is the detector area, and d is the duty cycle defined as d=W/P with W being the width of the pulse and P the pulse period.

#### 2.3.2. Range Observation Based on XCOM

XCOM is a revolutionary concept in deep space exploration, which utilizes X-ray to convey information [29]. As X-rays are high-energy light and have little attenuation in vacuum, they are suitable for deep space exploration. Besides, X-ray detectors can be quite small and energy-saving, which makes them suitable for long distance and long duration missions.

In this paper, the range between the spacecraft and a known location near the Earth is utilized as an observation to improve the performance of XNAV. For the range measurement, an X-ray circularly polarized ranging method based on XCOM has been proposed in our previous research, which will be illustrated in what follows.

Based on the idea of XCOM, we propose a circularly polarized ranging method (XCPolR) [20]. The proposed method utilizes circularly polarized X-ray as the ranging signal. The generation and detection of X-ray polarization signal can refer to the related references [30,31,32,33]. The polarized ranging signal can be expressed as:

(25) $$s(t)=\left\{S_{i}(A_{x},A_{y},\Psi (\text{r}(t)))\right\},\;i\in N,i<M$$

where r(t) is the ranging signal, M is the length of the ranging signal, Ψ(k) determines the polarization states to be either the left-hand circular polarization state or the right-hand circular polarization state. Ψ(k) is defined as:

(26) $$\Psi (k)=\left\{ \begin{array}{l} \pi /2+2n\pi \;k=1\\ -\pi /2+2n\pi \;k=0 \end{array}\right.\;n\in \text{Z}$$

**Figure 2:**
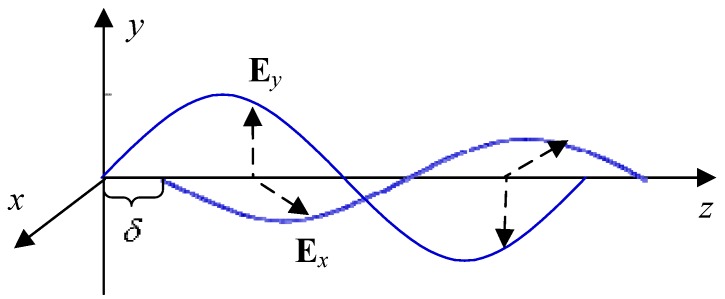
Polarized light.

When k=1, Ψ(k) represents the left-hand polarization state. Otherwise, it is the right-hand polarization state. $S_{i}(\cdot )$ is the Stokes parameter used to describe the polarization states, which is defined as:

(27) $$S(A_{x},A_{y},\delta )={\left[S_{0},S_{1},S_{2},S_{3}\right]}^{\text{T}}$$

(28) $$\left\{ \begin{array}{l} S_{0}=A_{x}^{2}+A_{y}^{2}\\ S_{1}=A_{x}^{2}-A_{y}^{2}\\ S_{2}=2A_{x}A_{y}cos\delta \\ S_{3}=2A_{x}A_{y}\sin\delta \end{array}\right.$$

where $A_{i}(i=x,y)$ is the intensity of the light vector components and δ is the phase error between the two orthogonal light vector components. These three parameters can be found in Figure 2. As shown in Figure 2, the two orthogonal light vector components, $E_{x}$ and $E_{y}$, are defined as:

(29) $$\left\{ \begin{array}{l} E_{x}=A_{x}\cos(\omega t+\phi_{x})\\ E_{\text{y}}=A_{y}\cos(\omega t+\phi_{y}) \end{array}\right.$$

where $\phi_{i}(i=x,y)$ are the phases of the components, and ω is the angular frequency.

In essence, the X-ray range measurement is a two-way measurement, whose procedure can be summarized as:

(1) At the emitter, modulate the X-ray signal with the binary ranging code using the circular polarization modulation and send the modulated X-ray signal to the receiver.
(2) Through the propagation in space channel, the X-ray ranging signal is received by the receiver and the signal is demodulated to recover the binary ranging sequence.
(3) Regenerate the downlink signal based on the recovered uplink signal and modulate the regenerated signal with the circular polarization modulation. Then, the downlink signal is sent back to the emitter.
(4) Receive the downlink signal at the emitter and demodulate the signal. Correlate the received signal with the local ranging sequence to obtain the two-way time delay. The two-way range can be calculated based on the elapsed time.

Let the coordinates of the known location near the Earth be $r_{0}={\left[x_{0},y_{0},z_{0}\right]}^{\text{T}}$ and D be the range measured by XCOM based XCPolR. Then, the range measurement can be expressed as:

(30) $$D=||r-r_{0}||$$

The estimated range measurement can be defined as:

(31) $$\tilde{D}=||\tilde{r}-r_{0}||$$

Then, the observation residual can be written as:

(32) $$\begin{array}{l} z_{r}=D-\tilde{D}+v_{r}\\ \;\approx \frac{{\tilde{r}}^{\text{T}}}{\tilde{r}}\delta r+v_{r}\\ \;=H_{r}\delta X+v_{r} \end{array}$$

where $\tilde{r}=||\tilde{r}||$, and $H_{r}$ is the range observation matrix defined as:

(33) $$H_{r}=\left[\begin{array}{cccc} \frac{{\tilde{r}}^{\text{T}}}{\tilde{r}} & 0 & 0 & 0 \end{array}\right]$$

and $v_{r}$ is the ranging jitter. According to the analysis in Reference [20], the ranging jitter mainly comes from the acquisition of the ranging signal. The ranging jitter can be evaluated by the standard variance of the delay measurement error expressed by:

(34) $$\begin{array}{l} \sigma_{r}=\frac{c\sigma_{\tau }}{2}\\ \;=\frac{c}{2}\cdot \frac{T_{rc}}{4\sqrt{2}T_{cor}}\cdot \sqrt{\frac{N_{0}}{P_{rc}}}\\ \;=\frac{cT_{rc}}{8\sqrt{SNR}T_{cor}} \end{array}$$

where $T_{rc}$ is the duration of one slot of the ranging signal, $N_{0}/2$ is the two-side power spectral density of the noise in ranging signal, $P_{rc}$ is the power of the clock component of the ranging signal, $\omega_{rc}$ is the angular frequency of the ranging clock component, and $T_{cor}$ is the correlation time when the ranging signal is acquired. The Signal-to-Noise Ratio of the ranging signal, *SNR*, is defined as:

(35) $$SNR=\sqrt{\frac{2P_{rc}}{N_{0}}}$$

### 2.4. Kalman Filtering Algorithm for State Estimation

As for the proposed method, both the pulsar timing observation and the range measurement are considered in the position determination of a spacecraft. In previous sections, the pulsar timing observation and the range measurement have been analyzed. Here, we would like to establish the observation equation based on the pulsar observation and the range measurement.

Assume two pulsars are observed and one range measurement is conducted. The observation equation can be established as:

(36) $$\begin{array}{l} Z={\left[H_{p1}^{\text{T}},H_{p2}^{\text{T}},H_{r}^{\text{T}}\right]}^{\text{T}}\delta X+V\\ \;=H\delta X+V \end{array}$$

where $H_{p1}^{\text{T}}$ and $H_{p2}^{\text{T}}$ are the pulsar observation matrices defined by Equation (21), and V is the observation noise. The covariance matrix of the observation noise is defined as:

(37) $$R=E(VV^{T})=diag(\sigma_{1}^{2},\sigma_{2}^{2},\sigma_{r}^{2})$$

where $\sigma_{i}(i=1,2)$ is the pulsar timing observation noise related to pulsar parameters and $\sigma_{r}$ is the ranging jitter determined by Equation (34).

Based on the state transition equation and the observation equation, the Kalman filtering process could be established:

(1) State prediction

As indicated by the state transition equation, the one-step state prediction can be expressed as:

(38) $${\hat{X}}_{k\text{+}1}=\Phi_{k}{\tilde{X}}_{k}+w_{k}$$

where $\Phi_{k}$ is the state transition matrix, ${\tilde{X}}_{k}$ is the optimal estimation of the system state at k, and ${\hat{X}}_{k+1}$ is the one-step prediction of the system state at k+1. $w_{k}$ is the process noise whose covariance matrix is defined as $Q_{w}=E(w_{k}w_{k}^{\text{T}})$.

The covariance matrix of the prediction error, ${\hat{P}}_{k+1}$, can be written as:

(39) $${\hat{P}}_{k+1}=\Phi_{k}{\tilde{P}}_{k}\Phi_{k}^{\text{T}}+Q_{W}$$

where ${\tilde{P}}_{k}$ is the covariance matrix of the prediction error at k.

(2) Filtering gain calculation

The filtering gain can be calculated as

(40) $$K_{k+1}={\hat{P}}_{k+1}H^{\text{T}}{\left(H{\hat{P}}_{k+1}H^{\text{T}}+R\right)}^{-1}$$

(3) Estimate the system state

The optimal estimation of the state at k+1, denoted by ${\tilde{X}}_{k\text{+}1}$, can be calculated by

(41) $${\tilde{X}}_{k\text{+}1}={\hat{X}}_{k\text{+}1}+K_{k+1}Z$$

where ${\hat{X}}_{k\text{+}1}$ is the predicted state.

(4) Update the prediction error covariance

(42) $${\tilde{P}}_{k+1}=(I-K_{k+1}H){\hat{P}}_{k+1}{\left(I-K_{k+1}H\right)}^{\text{T}}+K_{k+1}RK_{k+1}^{\text{T}}$$

## 3. Results and Discussion

### 3.1. Simulation Conditions

The area of the X-ray detector is set to 10,000 cm^2^ and the background flux intensity is set to be 0.0050 ph/cm^2^/s. The parameters of the pulsars used in the simulation are listed in Table 1.

The initial error is set to:

(43) $$\delta X={\left[800\text{ m},\;800\text{ m},\;800\text{ m},\;4\;\text{m}/\text{s},\;4\text{ m}/\text{s},\;4\;\text{ m}/\text{s}\right]}^{\text{T}}$$

The covariance matrix of the process noise is set to:

(44) $$Q_{w}={\left[{\left(0.5\text{ m}\right)}^{2},{\left(0.5\text{ m}\right)}^{2},{\left(0.5\text{ m}\right)}^{2},{\left(0.1\;\text{m}/\text{s}\right)}^{2},{\left(0.1\;\text{m}/\text{s}\right)}^{2},{\left(0.1\;\text{m}/\text{s}\right)}^{2}\right]}^{\text{T}}$$

The initial covariance matrix of the prediction error, $P_{0}$, is set to:

(45) $$P={\left[{\left(800\text{ m}\right)}^{2},{\left(800\text{ m}\right)}^{2},{\left(800\text{ m}\right)}^{2},{\left(4\text{ m}/\text{s}\right)}^{2},{\left(4\;\text{ m}/\text{s}\right)}^{2},{\left(4\;\text{ m}/\text{s}\right)}^{2}\right]}^{\text{T}}$$

The covariance matrix of the observation noise is calculated based on Equations (24) and (34), respectively.

The accurate orbits are simulated by the Satellite Tool Kit (STK), which is a professional software for simulating the design, test, and operation of space missions. The High-Precision Orbit Propagator (HPOP) is utilized to generate the Mars orbit in this paper. The parameters of the designed orbit are set as follows: (1) the orbit is a circular orbit with a radius of 46,792.48 km; (2) the orbit inclination is 45°. For simplicity, this orbit is named as “Orbit 1”.

The position error and velocity error can be evaluated by the following equations:

(46) $$\left\{ \begin{array}{l} delt\_r=\sqrt{{\left(r-\tilde{r}\right)}^{\text{T}}(r-\tilde{r})}\\ delt\_v=\sqrt{{(\dot{r}-\dot{\tilde{r}})}^{T}(\dot{r}-\dot{\tilde{r}})} \end{array}\right.$$

**Table 1:** Pulsar parameters.

Pulsars	Period/(s)	Right Ascension/(Rad)	Declination/(Rad)	Flux Intensity/(ph/cm^2^·s)	Pulse Width/(s)	Pulsed Fraction/(%)
B1937 + 21	1.558e − 3	5.1472	0.3767	4.99e − 5	3.82e − 5	86
B1821 − 24	3.050e − 3	4.8194	−0.4341	1.93e − 4	5.50e − 5	98
B0531 + 21	3.339e − 2	1.45967	0.384688	1.54	3.0e − 3	70

### 3.2. State Estimation Based on Kalman Filtering

In this section, the Kalman filtering results obtained based on the proposed method are presented and comparisons are made between the proposed method and the traditional pulsar observation method that estimates the system state by observing three pulsars.

**Figure 3:**
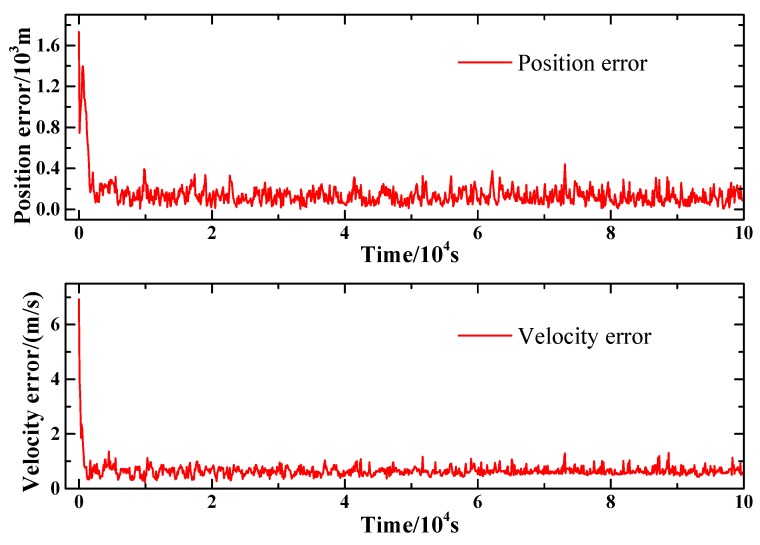
Position and velocity estimation error of the proposed method.

The Mars orbit used in the simulation is Orbit 1, whose parameters has been given in Section 3.1. The filtering interval is set to 100 s and the pulsar timing observation interval is set to 800 s. The first two pulsars listed in Table 1 are utilized to achieve pulsar timing observation. The SNR of the ranging signal is set to be 0 dB and the slot duration, $T_{rc}$, is set to be 1e−7 s. Figure 3 presents the state estimation results of the proposed method. It can be seen from the figures that the position and velocity error can converge to a low level, which demonstrates the feasibility of the proposed method.

**Figure 4:**
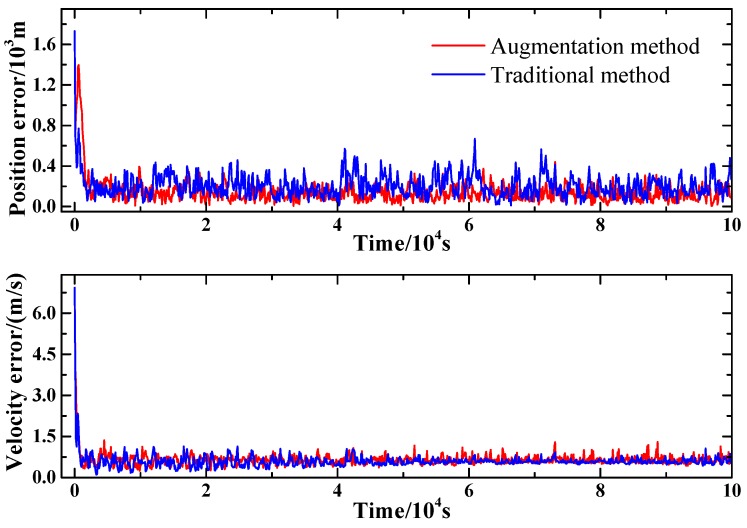
Comparison of the proposed method with the traditional method.

The comparison between the proposed method and the traditional method with only pulsar observation are conducted. For the proposed method, the range measurement, together with the timing observation of the first two pulsars listed in Table 1 are utilized for the state estimation. For the traditional method, all three pulsars listed in Table 1 are utilized for the state estimation. Figure 4 presents the comparison of the proposed method with the traditional method. Though the proposed method shows little superiority in velocity estimation compared with the traditional method, the proposed method does show better performance in position estimation under the same condition. The mean position estimation error of the traditional method remains at a level of 200 m, while the proposed method obtains a mean position error of 124 m. Besides, the standard variance of the position estimation error of the traditional method is 102.7 m, which is much larger than that of the proposed method whose standard variance is 65 m. This indicates that the proposed method shows better stability in the estimation of the position.

### 3.3. Impact of Pulsar Observation Interval on Filtering

To analyze the influence of the pulsar observation time on the filtering, a numerical experiment is conducted. The orbit used in the simulation is Orbit 1. The first two pulsars listed in Table 1 are utilized to achieve pulsar timing observation. The SNR of the ranging signal is set to be 0 dB and the slot duration is set to $T_{rc}=1e-7\;s$. The filtering interval is set to be 100 s.

**Figure 5:**
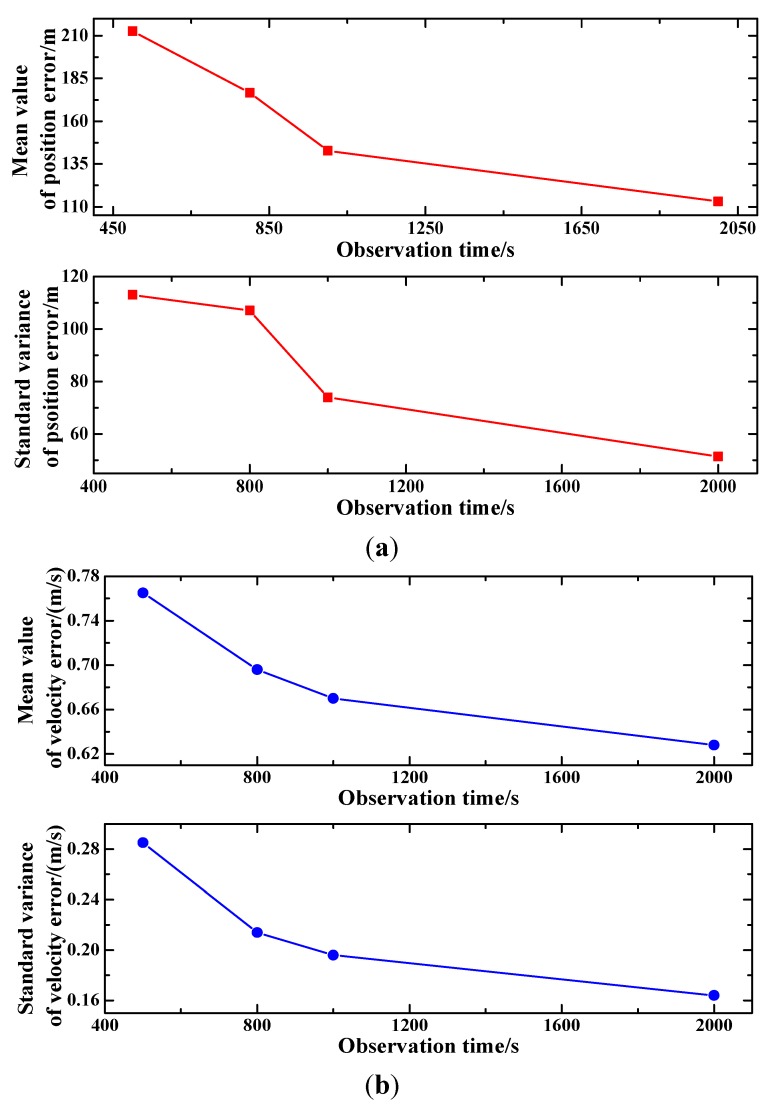
Influence of observation interval on filtering results. (**a**) Mean and standard variance of position error under different observation intervals; (**b**) Mean and standard variance of velocity error under different observation intervals.

Set the pulsar observation time intervals to be 500 s, 800 s, 1000 s, and 2000 s, respectively, and conduct the filtering of the proposed method under different observation time intervals. Then, the mean values and standard variances of the position and velocity estimation errors are calculated and plotted in Figure 5. Figure 5a presents the impact of observation time intervals on position estimation and Figure 5b is the velocity estimation under different observation time intervals. As shown in Figure 5a, with the increasing pulsar observation time intervals, both the mean and standard variance of the position estimation error decrease. It can also be observed from Figure 5b that the mean value and standard variance of the velocity show a similar tendency with the increasing observation time intervals as that of the position estimation. The reason for the phenomenon is that the noise of the pulsar observation is closely related to the observation time. As pulsar signals are weak, the observed signal is accumulated over a certain duration to strength the pulsar signal. The longer the observation time is, the higher the SNR of the pulsar signal is. Thus, long pulsar observation time could mitigate the observation noise, and consequently improve the performance of the position and velocity estimation.

### 3.4. SNR of Ranging Signal on Filtering

As the range measurement is utilized as the observation variable in the proposed method, the accuracy of the range measurement would have an influence on the filtering performance. On the other hand, the range measurement accuracy is highly related to the SNR of the ranging signal. Thus, in this section, we analyze the impact of the ranging signal SNR on the position and velocity estimation.

**Figure 6:**
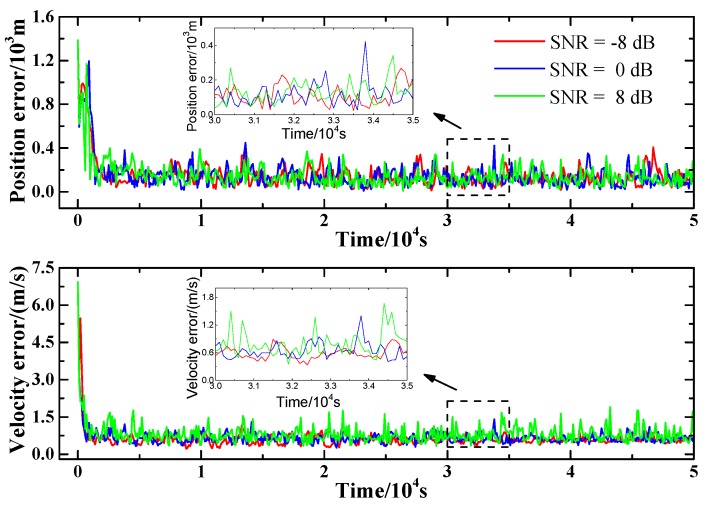
Influence of SNR on position and velocity estimation.

In the simulation, the initial conditions are as follows: (1) the orbit utilizes Orbit 1; (2) the filtering interval is set to 100 s and the pulsar observation interval is set to 1000 s, respectively; (3) SNR of the ranging signal is set to be −8 dB, 0 dB, 8 dB, respectively. Based on the initial conditions, the filtering of the proposed method is conducted, and the results are plotted in Figure 6. As shown in the figure, the influence of SNR of ranging signal on position and velocity estimation is minor. This is because of the differences in level of noises of the pulsar observation and the ranging measurement. The noise level of the range measurement is much lower than that of the pulsar observation. Thus, when the noise of the range measurement is influenced by the variation of the SNR of the ranging signal, the influence of the change in the range measurement noise is minor because the change is small relative to the high level of the pulsar observation noises.

### 3.5. Filtering Algorithm on Different Orbits

Different Mars orbits are used in this section to analyze the performance of the proposed method. Besides of Orbit 1 introduced in Section 3.1, we introduce another orbit, which is a stationary orbit of Mars. Its parameters are as follows: (1) the orbit is a circular orbit with a radius of 20,429.893717 km; (2) the inclination of the orbit is 0.014°. For simplicity, this orbit is named as “Orbit 2”. Other parameters are set as follows: (1) the filtering interval is set to 100 s and the pulsar observation time is set to 1000 s; (2) The SNR of the ranging signal is set to be 0 dB and the slot duration, $T_{rc}$, is set to be 1e−7 s; (3) the first two pulsars in Table 1 are utilized for pulsar observation.

**Figure 7:**
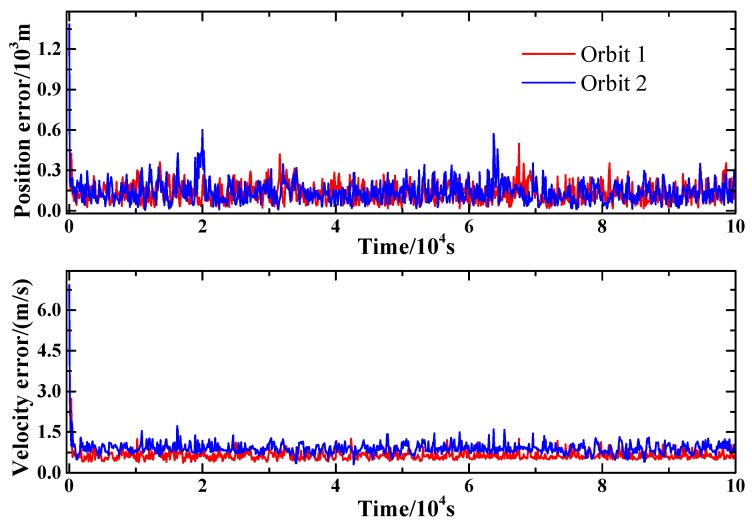
Filtering performance in different orbits.

Figure 7 presents the filtering results of the proposed method in different orbits. As shown in Figure 7, the performance of the position and velocity estimation on Orbit 1 and Orbit 2 shows much similarity. This indicates that the proposed method applies not only to the high Mars orbits, but also to the geostationary Mars orbits.

## 4. Conclusions

The lack of accuracy of XNAV has become a huge obstacle for its further development and application. In this paper, a novel XNAV augmentation method is documented, aiming at improving the XNAV accuracy. The proposed method utilizes X-ray ranging as the extra observation, together with traditional pulsar timing observation, to achieve the position and velocity estimation. The performance of the proposed method was verified by numerical experiments. It is demonstrated that the proposed method shows better performance than the traditional pulsar observation method in position and velocity estimation. Numerical experiments also indicated that the performance of the proposed method was influenced by several parameters, such as the pulsar observation interval, the SNR of the ranging signal, *etc*. Two different orbits were utilized to verify the feasibility of the proposed method on different types of orbits.
